# 1375. Professional Status of Infectious Disease Specialists in Korea: A Nationwide Cross-sectional Study

**DOI:** 10.1093/ofid/ofab466.1567

**Published:** 2021-12-04

**Authors:** Bongyoung Kim, Byung Wook Eun, Eunjung Lee, Tae Hyong Kim, Suyeon Park, Se Yoon Park

**Affiliations:** 1 Hanyang University College of Medicine, Seongdong-gu, Seoul-t’ukpyolsi, Republic of Korea; 2 Eulji University Eulji General Hospital, Seoul, Seoul-t’ukpyolsi, Republic of Korea; 3 Soonchunhyang University Seoul Hospital, Seoul, Korea, Seoul, Seoul-t’ukpyolsi, Republic of Korea; 4 Division of Infectious Diseases, Department of Internal Medicine, Soonchunhyang University Seoul Hospital, Soonchunhyang University College of Medicine, Seoul, Korea, Seoul, Seoul-t’ukpyolsi, Republic of Korea; 5 Soonchunhyang University Seoul Hospital, Seoul, Seoul-t’ukpyolsi, Republic of Korea

## Abstract

**Background:**

Emergence of more antimicrobial-resistant pathogens and repeated occurrence of infectious disease (ID) outbreaks highlight the importance of ID specialists. This study aimed to assess the working status of ID specialists and identify problems faced by ID professionals in Korea.

**Methods:**

An online-based survey was conducted over 11 days (from December 17–27, 2020), targeting all active adult (n=281) and pediatric (n=71) ID specialists in Korea (total=352). An online-based survey link was forwarded to them via text messages and e-mails by the office of the Korean Society of Infectious Diseases and the Korean Society of Pediatric Infectious Diseases. Questions regarding the practice areas of the specialists were divided into five categories: (1) clinical practices of outpatient care, inpatient care, and consultations; (2) infection control; (3) antibiotic stewardship; (4) research; and (5) education and training. We investigated the weekly time-use patterns for these areas of practice.

**Results:**

A total of 144/281 (51.2%) adult ID specialists and 51/71 (71.8%) pediatric ID specialists participated in the survey. Among them, 144 (73.8%) respondents were involved in all practice categories investigated. The most common practice area was outpatient service (93.8%), followed by consultation (91.3%) and inpatient service (87.7%)(Table 1). Specialists worked a median of 57 (interquartile range: 50–65) hours weekly: patient care, 29 (14–37) hours; research 11 (5–19) hours; infection control 4 (2–10) hours; antibiotic stewardship, 3 (1–5) hours; and education/training, 2 (2–6) hours (Table 2).

**Conclusion:**

We identified areas of practice and patterns of time use among adult and pediatric ID specialists in Korea. Most experts were in charge of all necessary areas (including treatment, education, research, infection control, and antibiotic stewardship) in medical institutions with limited resources. It is expected that these problems can be solved by appropriately compensating individuals and medical institutions for their invisible activities (including infection control and antibiotic stewardship) and by securing additional human resources.

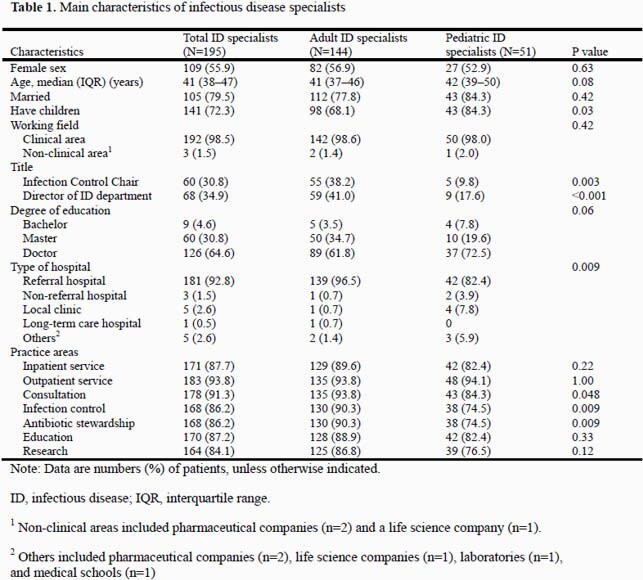

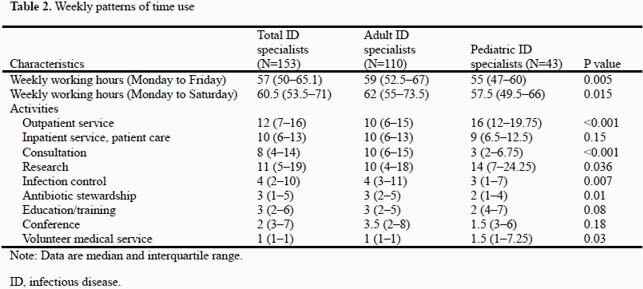

**Disclosures:**

**All Authors**: No reported disclosures

